# The association between serum microbial DNA composition and symptoms of depression and anxiety in mood disorders

**DOI:** 10.1038/s41598-021-93112-z

**Published:** 2021-07-07

**Authors:** Sang Jin Rhee, Hyeyoung Kim, Yunna Lee, Hyun Jeong Lee, C. Hyung Keun Park, Jinho Yang, Yoon-Keun Kim, Yong Min Ahn

**Affiliations:** 1 Department of Neuropsychiatry, Seoul National University Hospital, Seoul, Republic of Korea; 2 Department of Psychiatry, Seoul National University College of Medicine, Seoul, Republic of Korea; 3 Department of Psychiatry, National Cancer Center; Division of Cancer Management Policy, National Cancer Center, Goyang, Republic of Korea; 4 MD Healthcare Inc., Seoul, Republic of Korea; 5 Institute of Human Behavioral Medicine, Seoul National University Medical Research Center, Seoul, Republic of Korea; 6 Present Address: Department of Psychiatry, Inha University Hospital, Incheon, Republic of Korea; 7 Present Address: Department of Psychiatry, Kosin University Gospel Hospital, Busan, Republic of Korea; 8 Present Address: Department of Psychiatry, Asan Medical Center, Seoul, Republic of Korea

**Keywords:** Depression, Translational research

## Abstract

There is increasing evidence supporting the association between gut microbiome composition and mood disorders; however, studies on the circulating microbiome are scarce. This study aimed to analyze the association of the serum microbial DNA composition with depressive and anxiety symptoms in patients with mood disorders. The sera of 69 patients with mood disorders, aged from 19 to 60, were analyzed. Bacterial DNA was isolated from extracellular membrane vesicles and, subsequently, amplified and quantified with specific primers for the V3–V4 hypervariable region of the 16S rDNA gene. Sequence reads were clustered into Operational Taxonomic Units and classified using the SILVA database. There were no significant associations between alpha diversity measures and the total Hamilton depression rating scale (HAM-D) or Beck anxiety inventory (BAI) scores. Only the weighted UniFrac distance was associated with the total HAM-D score (F = 1.57, *p* = 0.045). The *Bacteroidaceae* family and *Bacteroides* genus were negatively associated with the total HAM-D score (β =  − 0.016, *p* < 0.001, q = 0.08 and β =  − 0.016, *p* < 0.001, q = 0.15, respectively). The *Desulfovibrionaceae* family and *Clostridiales* Family XIII were positively associated with the total BAI score (β = 1.8 × 10^−3^, *p* < 0.001, q = 0.04 and β = 1.3 × 10^−3^, *p* < 0.001, q = 0.24, respectively). Further studies with larger sample sizes and longitudinal designs are warranted.

## Introduction

The gut microbiome is a dynamic ecosystem of microorganisms and their genes that inhabit in the gut^1^. Research has revealed evidence of the “gut-brain axis”, a bidirectional interaction between gut microbiome and the central nervous system via endocrine, neuronal, and immune pathways^2^. Therefore, there is increasing interest in the role of the gut microbiome in psychiatry research^3^. Studies have mainly focused on the gut microbiome and its ability to differentiate disorders from healthy states or differentiate between disorders, including mood disorders^3–5^.

Depression and anxiety are common symptoms in mood disorders. Recent reports have suggested that the effects of microbiome in mood disorders might be a more general phenomenon related to psychiatric symptoms than specific to diagnosis^6,7^. Investigating the relationship between these symptoms and the microbiome composition will enable us to compare with previous reports focusing on the composition differences between diseased states and healthy controls (HC), to see if the microbial composition that differed between diseased states and HC, are actually related to psychiatric symptoms^7^. It will also give us further knowledge of the biological basis of these symptoms.

Some studies on the gut microbiome have shown an association between microbial composition and psychiatric symptoms, with controversial results^6–15^. Recently a systematic review summarizing the results of gut microbiota analysis in anxiety and depression was published^16^. Most of these studies were based on simple correlation analyses^6,10–14^ and did not control significant covariates, including demographics that were associated with these symptoms. Studies should control significant covariates and consider the nature of relative microbial abundances.

Moreover, no study, to our knowledge, has investigated the relationship between the circulating microbial DNA and psychiatric symptoms. Recently, researchers have expanded their focus to microbiome of other sites such as blood. Blood has its advantages as it is easy to obtain, and it reflects systemic conditions. The studies from diseases such as chronic kidney disease, diabetes, and alcoholic liver disease have revealed the potential of microbial DNA in the blood as a circulating biomarker, as the composition was different when compared to HC^17–19^. Microbe-derived extracellular vesicles (EVs) are 20–200 nm sized molecules secreted by bacteria that contain genomic DNA fragments^20^. These EVs are absorbed in the blood as they can pass through the mucus membrane^20^. The analysis of microbe-derived EVs has further advantages as these EVs imply that the microbiome is metabolically or pathologically active, and may represent the major constitutes of microbes in the body^21–23^.

We previously analyzed the relationship of the serum microbial DNA composition in patients with major depressive disorder (MDD), bipolar disorder (BD), and HC^4^. The present analysis was based on our previous data, focusing on the relationship between the serum microbial DNA composition and psychiatric symptoms for patients with mood disorders. We explored whether serum microbial DNA diversity and individual taxa had a significant association with depressive and anxiety symptoms.

## Results

### Demographic and clinical characteristics

The demographic and clinical characteristics of the study population are shown in Table 1. Further associations with psychiatric symptoms are shown in Table 2. Only exercise was associated with the total Hamilton depression rating scale (HAM-D) score (t =  − 2.05, *p* = 0.044) and total Beck anxiety inventory (BAI) score (t =  − 3.69, *p* < 0.001) and was controlled as a covariate for the following analysis.

**Table 1:** Demographic and clinical characteristics of the study population (n = 69).

Age, mean ± SD, years	39.6 ± 12.0
**Sex**	
Male, *n* (%)	18 (26.1)
BMI, mean ± SD, kg/m^2^	24.2 ± 4.1
Exercise, *n* (%)	30 (43.5)
Current smoker, *n* (%)	12 (17.4)
Alcohol use, *n* (%)	30 (43.5)
HAM-D total score, mean ± SD	6.13 ± 5.08
BAI total score, mean ± SD	8.71 ± 10.31
YMRS total score, mean ± SD	2.48 ± 3.31
**Mood disorder type**	
MDD, *n* (%)	29 (42.0)
BD, *n* (%)	40 (58.0)
**Medication**	
Antidepressant use, *n* (%)	31 (44.9)
Anticonvulsant or lithium use, *n* (%)	33 (47.8)
Antipsychotics use, *n* (%)	45 (65.2)

*SD* standard deviation, *BMI* body mass index, *HAM-D* Hamilton depression rating scale, *BAI* Beck anxiety inventory, *YMRS* Young mania rating scale, *MDD* major depressive disorder, *BD* bipolar disorder.

**Table 2:** Association of demographic and clinical variables with psychiatric symptoms (n = 69).

	HAM-D total score	BAI total score
Age	t = − 1.27, *p* = 0.21	t = − 0.64, *p* = 0.52
Sex	t = 0.07, *p* = 0.94	t = 1.76, *p* = 0.09
BMI	t = − 0.41, *p* = 0.68	t = − 0.72, *p* = 0.48
Exercise	**t = ** − **2.05,** *p*** = 0.044**	**t = ** − **3.69,** *p*** < 0.001**
Current smoker	t = − 1.32, *p* = 0.20	t = 0.36, *p* = 0.72
Alcohol use	t = − 0.49, *p* = 0.63	t = − 0.26, *p* = 0.80
Mood disorder type	t = 0.32, *p* = 0.75	t = 0.15, *p* = 0.88
Antidepressant use	t = − 0.70, *p* = 0.48	t = − 0.69, *p* = 0.49
Anticonvulsant or lithium use	t = 0.63, *p* = 0.53	t = 0.69, *p* = 0.49
Antipsychotics use	t = 1.05, *p* = 0.30	t = 1.10, *p* = 0.28

*HAM-D* Hamilton depression rating scale, *BAI* Beck anxiety inventory, *BMI* body mass index.

Pearson’s correlation were performed for continuous variables and t-tests were performed for categorical variables, boldface values are statistically significant at *p* < 0.05.

### Diversity analysis

There was no significant association between the alpha diversity measures and the total HAM-D or total BAI scores (Table 3). For beta diversity measures, the weighted UniFrac distance was associated with the total HAM-D score (F = 1.57, *p* = 0.045). However, for other measures, there was no significant association with the total HAM-D scores or the total BAI scores (Table 4).

**Table 3:** Association of serum alpha-diversity with psychiatric symptoms (n = 69).

Parameter	HAM-D total score	BAI total score
Observed OTU^†^, mean ± *SD*	β = − 0.001, t = − 0.10, *p* = 0.92	β = 0.004, t = 0.82, *p* = 0.42
Chao-1 index^†^, mean ± *SD*	β = 0.001, t = 0.16, *p* = 0.88	β = 0.005, t = 0.98, *p* = 0.33
Inverse Simpson index, mean ± *SD*	β = 0.148, t = 0.55, *p* = 0.59	β = 0.090, t = 0.64, *p* = 0.52
Shannon index^‡^, mean ± *SD*	β = 0.138, t = 0.32, *p* = 0.75	β = 0.206, t = 0.94, *p* = 0.35

*HAM-D* Hamilton depression scale, *BAI* Beck anxiety inventory, *OTU* operational taxonomic unit, *SD* standard deviation.

Linear regression was performed with adjustment for exercise.

^†^Due to skewed distribution, log-transformation was performed before analysis.

^‡^Due to skewed distribution, exponential-transformation was performed before analysis.

**Table 4:** Association of serum beta-diversity with psychiatric symptoms (n = 69).

Parameter	HAM-D total score	BAI total score
Bray–Curtis dissimilarity	F = 1.18, *p* = 0.12	F = 1.13, *p* = 0.18
Unweighted UniFrac distance	F = 0.93, *p* = 0.72	F = 1.12, *p* = 0.12
Weighted UniFrac distance	**F = 1.57, *p* = 0.045**	F = 0.99, *p* = 0.41

*PERMANOVA* permutational analysis of variance, *HAM-D* Hamilton depression scale, *BAI* Beck anxiety inventory.

PERMANOVA was performed with adjustment for exercise, boldface value is statistically significant at *p* < 0.05.

### Taxonomic analysis

A total of 73 families and 137 genera were utilized in the taxonomic analysis. The *Bacteroidaceae* family and *Bacteroides* genus (in the *Bacteroidaceae* family) were negatively associated with the total HAM-D score (β =  − 0.016, *p* < 0.001, q = 0.08 and β =  − 0.016, *p* < 0.001, q = 0.15, respectively). The *Desulfovibrionaceae* family and *Clostridiales* Family XIII were positively associated with the total BAI score (β = 1.8 × 10^−3^, *p* < 0.001, q = 0.04 and β = 1.3 × 10^−3^, *p* < 0.001, q = 0.24, respectively). There was no significant genus associated with the total BAI score. However when considering multiple comparison, only the *Bacteroidaceae* and *Desulfovibrionaceae* family were statistically significant.

**Figure 1:**
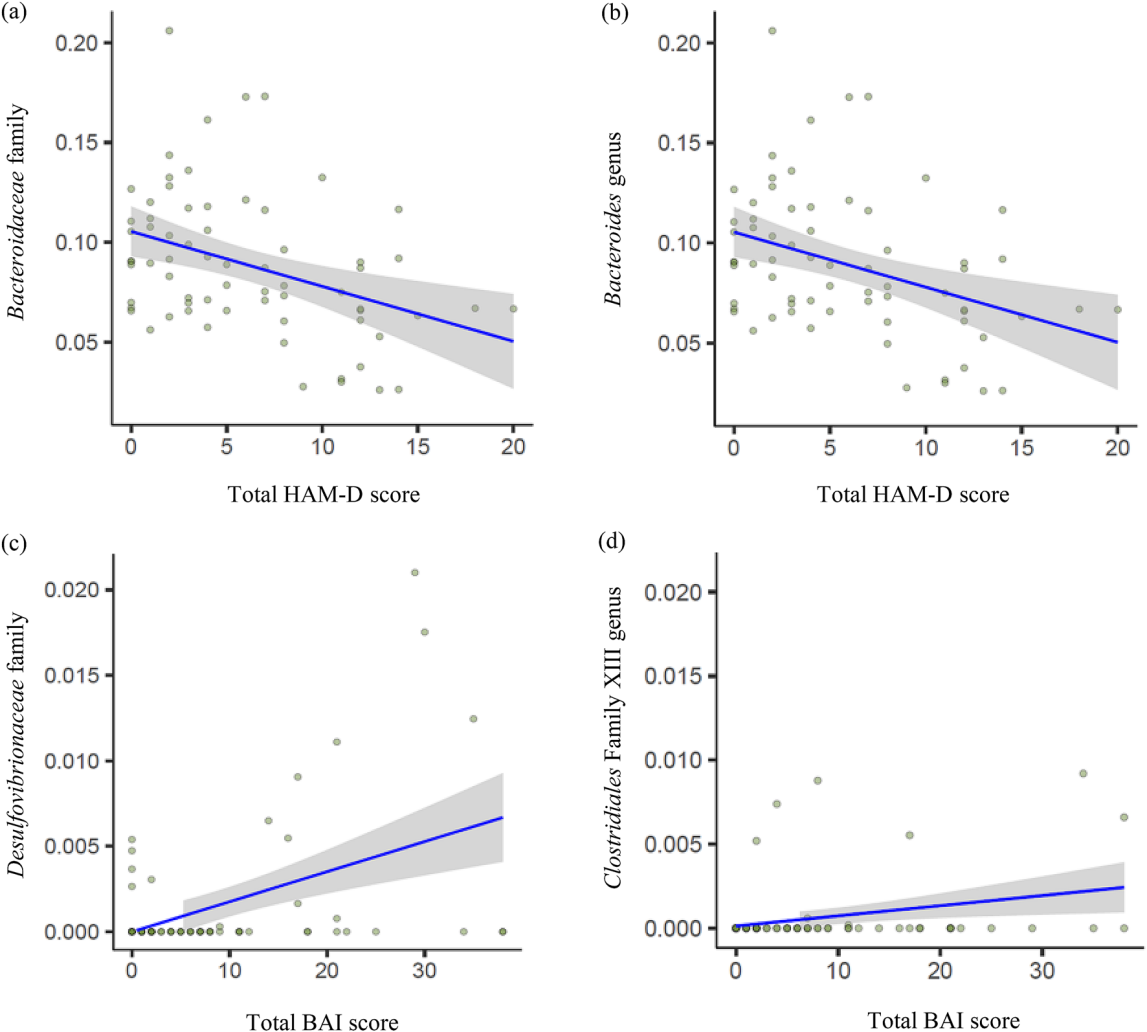
Abundances of significant taxa associated with psychiatric symptom scores. Significant taxa were plotted as log-transformed relative abundances. The figure was generated using R version 3.6.2 (URL link: https://www.R-project.org/). Abbreviations: HAM-D = Hamilton depression rating scale, BAI = Beck anxiety inventory.

The results are shown in Table 5, and the association between significant taxa and psychiatric symptom scores is plotted in Fig. 1.

**Table 5:** Association of individual serum microbial taxa with psychiatric symptoms.

Variable	Domain	Feature	Coefficient (β)	SE	N	Non zero N	*p* value	q-value
HAM-D	Family	Bacteria|Bacteroidetes|Bacteroidia| Bacteroidales|Bacteroidaceae	** − 0.016**	**4.3 × 10**^**−3**^	**69**	**69**	**5.4 × 10**^**−4**^	**0.08**
HAM-D	Genus	Bacteria|Bacteroidetes|Bacteroidia| Bacteroidales|Bacteroidaceae|Bacteroides	** − **0.016	4.3 **×** 10^−3^	69	69	5.4 **×** 10^−4^	0.15
BAI	Family	Bacteria|Proteobacteria|Deltaproteobacteria| Desulfovibrionales|Desulfovibrionaceae	**1.8 × 10**^**−3**^	**4.8 × 10**^**−4**^	**69**	**15**	**2.9 × 10**^**−4**^	**0.04**
BAI	Family	Bacteria|Firmicutes|Clostridia| Clostridiales|Family XIII	1.3 **×** 10^−3^	4.2 **×** 10^−4^	69	9	3.2 **×** 10^−3^	0.24

*SE* standard error, *HAM-D* Hamilton depression scale, *BAI* Beck anxiety inventory.

Analysis done with log-transformed relative abundance using MaAsLin2 (Multivariate association with linear models). Taxa with *p* value < 0.05 and q-value (Benjamini–Hochberg false discovery rate corrected) < 0.25 are shown. Boldfaces are statistically significant at q-value < 0.10 considering multiple comparison.

### Functional analysis

The abundances of a total of 275 unique KEGG Orthology pathways were predicted. However, after considering multiple comparisons, no statistically significant pathway was found to be associated with the total HAM-D or total BAI scores.

## Discussion

This study was the first to compare the serum microbial DNA composition and psychiatric symptom severities in mood disorders. There was no significant association between alpha diversity measures and the total HAM-D and BAI scores. Only the weighted UniFrac distance was significantly associated with the total HAM-D score. We revealed significant microbial families in the serum that were associated with the total HAM-D and BAI scores, however there were no significant KO pathways.

The present study showed no association of alpha diversity with either depressive or anxiety symptoms. However, we previously reported a higher serum alpha diversity, according to the Shannon index and inverse Simpson index, in both MDD and BD than in the HC^4^. Alpha diversity may help differentiate individuals with mood disorders from HC but may not depend on psychiatric symptoms. Additional studies that investigated the association between gut microbial alpha diversity and psychiatric symptoms have shown mixed results. There were studies reporting an inverse association between the Shannon index and anxiety symptoms in bipolar depression^10^, and the Faith’s phylogenetic diversity and depressive symptoms in MDD^7^. However other studies have reported negative results^8,9^.

We revealed in the present study that only the weighted UniFrac distance, which considers relative abundancies when calculating the distance, was associated with depressive symptoms. However, the weighted UniFrac distance was different between BD and HC and not between MDD and HC in our previous study^4^. On the other hand, a study of the gut microbiome revealed no association between beta diversity and depressive or anxiety symptoms in a study on a heterogeneous group of individuals with depression and anxiety and healthy control individuals^9^. This implies that serum microbial community structure reflects not only disease states, but also depressive symptoms. As the serum microbial community might be involved in the pathophysiology of depressive symptoms, researchers should try to reveal potential mechanisms.

When investigating individual taxa, the *Bacteroides* genus, which was negatively associated with depressive symptoms in the present study (although it lost statistical significance considering multiple comparison), did not show an association with mood disorders in our previous study^4^. Gut microbiome profiling studies that analyzed the associations with psychiatric symptoms in mood disorders were based on correlations^6,10–14^; some studies used regression^9,15^ or other statistical methods^7,8^. One study revealed that an Operational Taxonomic Unit (OTU) in the *Bacteroides* genus exhibited a positive association with composite scores for mental health from the Short Form Health Survey and a negative association with anxiety symptoms but no association with depressive symptoms in BD subjects^15^. No gut microbiome family or genus from these previous studies was replicated in our study with respect to the association of depressive or anxiety symptoms with the serum microbial DNA.

The *Bacteroides* genus in the gut has been repeatedly reported to be associated with depression; their abundance in depression has been found to be higher^11,24^ or lower^10,14^ when compared to that in controls. According to our study, they exhibited a negative association with depressive symptom severity in patients with mood disorders. Interestingly, there is evidence that the fecal *Bacteroides* has associations with known pathophysiologies of depression. It was demonstrated that the abundance of fecal *Bacteroides* is inversely correlated with brain signatures associated with depression, and that *Bacteroides* expresses GABA-producing pathways^25^. In the circulating microbiome, the *Bacteroides* genus has been associated with a decreased risk for type 2 diabetes^17^. Their level in non-alcohol-consuming control individuals was higher when compared to that in alcoholics^19^. It appears that the level of *Bacteroides* in the blood decreases under certain disease conditions^17,19^. Alcohol consumption was not associated with depressive symptoms in our study. Although we excluded individuals with diabetes, glucose intolerance, which is associated with depressive symptoms^26^, might be related to the circulating level of *Bacteroides* DNA.

The serum level of *Clostridiales* Family XIII DNA exhibited a positive association with anxiety symptoms in the present study (although it lost statistical significance considering multiple comparison). However, its level in the gut has been shown to be less abundant in generalized anxiety disorder (GAD) than in HC^27^. Therefore, this trend was relatively opposite for the serum and gut with respect to anxiety. Recently, an animal study revealed that when rats were treated with minocycline, the relative abundance of this family increased and that the abundance was associated with 3-OH butyrate^28^. However, although minocycline produces anti-anxiety effects^29^, it was shown to decrease depressive-like but not anxiety-like behavior in the animal study^28^.

*Bacteroides* are gram-negative, obligate anaerobes and are considered a major genus^30^. Several *Clostridiales* strains are associated with various psychiatric disorders^31^. Both are known to produce short chain fatty acids (SCFA)^30,31^. Especially, butyrate serves as a major energy source for colonocytes, and it has been reported to be beneficial for health as it exhibits anti-inflammatory properties. However the role of SCFA in the blood needs further exploration. The serum levels of SCFA were elevated in diabetes, and positively associated with adiposity measures^32,33^. On the other hand, SCFA were inversely correlated with inflammatory markers and endotoxin and had beneficial roles in those with cirrhosis^34^. The positive/negative role of circulating SCFA might also differ between depressive and anxiety symptoms, and concurrent measures of these SCFA are warranted in future studies^30,35^.

In our study, the *Desulfovibrionaceae* family DNA composition in the serum was positively associated with anxiety symptoms. Interestingly, the abundance of the *Desulfovibrionaceae* family in the gut has been shown to be lower in GAD patients than in HC^27^; therefore, the direction of the association was relatively opposite for the gut and serum with respect to anxiety. This family is composed of gram-negative anaerobes and is known to produce hydrogen sulfide^36^. Hydrogen sulfide penetrates through the cell membrane and inhibits butyrate oxidation^37^. Its toxicity leads to structural and functional changes in the gut barrier, provoking inflammation^37^. Although the role of *Desulfovibrionaceae* in the blood needs further investigation, the pro-inflammatory property of hydrogen sulfide could mediate the association between inflammation and anxiety severity^38^. Studies comparing other conditions have revealed that *Desulfovibrionaceae* was decreased in obese and total non-alcoholic fatty liver disease (NAFLD), but not in lean NAFLD, when compared to HC^39^, and decreased in chronic kidney disease when compared to HC^18^. Both studies were based on microbiome from the buffy coat, so direct comparison is limited.

However, studies on certain microbiome strains are primarily based on the role of their products in the gut; therefore, the link between the microbiome in the gut and blood, and the brain needs to be further investigated. Recently, a study showed that several gut microbes are correlated to several circulating microbes, indicating a link between gut and blood microbial composition^40^. Therefore, a change in the microbiome composition in the blood could reflect a change in the gut microbiome. However, the contradiction between previous data on the gut microbiome with respect to anxiety and the results from our present study on the circulating microbial DNA should be accounted. Anxiety states might increase translocation via dysfunction of the intestinal epithelium, resulting in opposite abundances in the gut and blood. Another mechanism to consider is that a change in the blood microbiome composition can stimulate inflammation. The cell walls of gram-negative bacteria are composed of lipopolysaccharide and that of gram-positive bacteria are composed of lipoteichoic acid, both which are known to provoke innate immune responses^41^. Therefore, a change in the composition of these bacteria could be associated with inflammation, which is a known factor for depression^42^ and anxiety^43^. However, we are unsure if the blood microbiome is from the gut, as oral sources can influence the blood microbiome^44^, and if they are intact microbiomes or DNA fragments from other areas of the body^45^.

This study has several limitations. First, as it was a cross-sectional study, causality could not be determined. Second, the sample size was small, and symptom severity was mild, which might have resulted in less statistical power and negative results including the functional analysis. The participants weren’t required to be currently in a depressive episode, so the lack of associations might have been due to the mild severity state. Replication studies with more severe patients are warranted. Third, analyses of the gut microbiome or other blood metabolites were not conducted. Concurrent measures would enable investigation of the specific mechanisms underlying the association between the microbial DNA and psychiatric symptoms. Fourth, the presence of other confounding factors, including diet and sampling time, could influence the microbial DNA composition^46,47^.

However, our study was the first to analyze the association between the serum microbial DNA composition and psychiatric symptom scores in patients with mood disorders. Unlike previous studies on mood disorders, we used MaAsLin2, a statistical approach used in microbial studies to account for the characteristics of microbial composition: high data dimensionality, sparsity, and mean–variance dependency^48^.

In conclusion, this study demonstrated that only the weighted UniFrac distance of serum microbial DNA was associated with depressive symptoms in mood disorders. Additionally, there were significant families that were associated with depressive and anxiety symptoms in patients with mood disorders. Further studies utilizing a larger sample size should consider longitudinal designs and evaluate the specific mechanisms underlying this association.

## Methods

### Study participants

Initially, 72 patients (42 with BD and 30 with MDD), with ages ranging from 19 to 60, were enrolled from the outpatient clinic of Seoul National University Hospital from 2015 to 2018. Diagnosis was made according to the criteria of the Diagnostic and Statistical Manual of Mental Disorders 4th or 5th version (DSM-IV or DSM-5) and was confirmed by the Mini-International Neuropsychiatric Interview (MINI). Depressive symptoms were assessed using the 17-item HAM-D^49^, and hypomanic symptoms were assessed using the 11-item Young mania rating scale (YMRS)^50^. These evaluations were administered by well-trained psychiatric research nurses. Anxiety symptoms were assessed using the 21-item BAI^51^. Information of exercise, current smoker, and alcohol use was based on self-reports asking the patients if they were regularly exercising, currently smoking, and regularly drinking alcohol. Body mass index (BMI) was calculated as weight (kg) divided by height (m) squared (kg/m^2^). Medication use was based on self-report. Three patients were excluded because of missing BAI data, resulting in 69 patients.

We excluded patients on antibiotics, antifungal agents, and steroids, and those diagnosed with hypertension, diabetes, cancer, rheumatoid diseases, gastrointestinal diseases including inflammatory bowel disorders, alcohol-use or other substance-use disorders, and eating disorders. For BD patients, those with hypomanic/manic/mixed episodes were excluded. Participants were allowed to continue taking other non-psychotropic medications. We did not exclude use of probiotics. Serum samples were collected from each participant.

The study was carried out in accordance with the latest version of the Declaration of Helsinki. The study design was reviewed by the Institutional Review Boards of Seoul National University Hospital (IRB No 1301-069-459). Informed consent of the participants was obtained after the nature of the procedures had been fully explained.

### Microbial DNA analysis

#### Extracellular vesicle isolation and DNA extraction

DNA extraction from EVs was performed as follows^4^. The serum from each participant was collected in serum separator tubes and centrifuged at 3000 rpm for 15 min at 4 °C. The supernatant was collected and stored at − 80 °C until analysis. Then, the supernatant was mixed with 1 × phosphate-buffered saline (pH 7.4, ML008-01, Welgene, Republic of Korea) and subsequently centrifuged at 10,000× *g* for 10 min at 4 °C. To isolate EVs, bacteria and foreign particles were thoroughly eliminated through sterilization of the supernatant using a 0.22 μm filter. The separated EVs were boiled for 40 min at 100 °C and centrifuged at 13,000 rpm for 30 min at 4 °C to eliminate the remaining floating particles and waste. EV DNA was extracted using the DNeasy Powersoil Kit (QIAGEN, Germany) and quantified using the QIAxpert system (QIAGEN).

#### Amplicon sequence analysis

The libraries were prepared using EV DNA PCR products according to the MiSeq System guide (Illumina, San Diego, CA, USA) and quantified using the QIAxpert system. We used the 16S_V3_F (5′-TCGTCGGCAGCGTCAGATGTGTATAAGAGACAGCCTACGGGNGGCWGCAG-3′) and 16S_V4_R (5′-GTCTCGTGGGCTCGGAGATGTGTATAAGAGACAGGACTACHVGGGTATCTAATCC-3′) primers, which are specific for the V3–V4 hypervariable region of the 16S rDNA gene, to amplify bacterial genomic DNA. Each amplicon was quantified, set at an equimolar ratio, pooled, and sequenced on a MiSeq system.

#### Microbial DNA bacterial composition analysis

Paired-end reads that matched the adapter sequences were trimmed using Cutadapt (version 1.1.6)^52^. The resulting FASTQ files were merged with CASPER^53^. The quality filter was applied with the Phred (Q) score, on the basis of the criteria described by Bokulich et al^54^. Any merged reads that were shorter than 350 bp or longer than 550 bp were discarded. A reference-based chimera searching method was performed to identify chimeric sequences, using VSEARCH with the SILVA gold database^55,56^. Next, the remaining sequence reads were clustered into OTUs via a de novo clustering method using VSEARCH at a 97% sequence similarity. We excluded OTUs that contained only one sequence in only one sample. The representative sequences of the OTUs were finally assigned taxonomy using UCLUST along with the SILVA 128 database [parallel_assign_taxonomy_uclust.py script on QIIME (version 1.9.1)] under default parameters^57^.

### Statistical analysis

The association of demographic and clinical variables with the psychiatric symptom scores was analyzed using Pearson’s correlation analysis for continuous variables and t-tests for categorical variables. Exercise was associated with both psychiatric symptom scores and was controlled for the following analysis.

Alpha diversity was calculated using the observed OTUs, Chao-1 indices, inverse Simpson indices, and Shannon indices. For skewed measures, normalization was performed before further analysis. Beta diversity was calculated by the Bray–Curtis dissimilarity and unweighted/weighted UniFrac distance matrix. Linear regression was performed for alpha diversity, and permutational analysis of variance (PERMANOVA) was performed for beta-diversity to analyze its association with psychiatric symptom scores.

Multivariate association with linear models (MaAsLin2) was performed to analyze the association between microbial DNA relative abundances (family and genus) and psychiatric symptom scores. MaAsLin2 performs a boosted, additive general linear model between microbial abundance and metadata^58^. Only taxa with a minimum abundance of 0.01%, present in at least 10% of the samples, were utilized for the analysis. Significant taxa were plotted as log-transformed relative abundances.

Finally, to obtain prediction of the functional pathway profiles, we used Tax4Fun for functional community profiling based on 16S rRNA data^59^. It annotates metabolic cycles and pathways in the Kyoto Encyclopedia of Genes and Genomes (KEGG)^60^. The psychiatric symptom score was the dependent variable, and the relative abundance of KEGG Orthology terms and exercise were the independent variables in the linear regression analysis.

All tests of significance, after controlling for the covariates, were two-sided; statistical significance was set at *p* < 0.05.

In line with default parameters and previous studies, taxa satisfying Benjamini–Hochberg false discovery rate at q < 0.25 was considered initially significant for MaAsLin2^58,61,62^. A more conservative q < 0.10 was used for statistical significance of multiple comparison in MaAsLin2 and functional analysis.

All statistical analyses were performed using R version 3.6.2.

## Data Availability

The datasets used and analyzed during the current study are available from the corresponding author on reasonable request.
